# Exploring the gut–kidney axis: possible connection between gastrointestinal and renal disorders in dromedary camels

**DOI:** 10.3389/fvets.2025.1689681

**Published:** 2025-11-14

**Authors:** Mohamed Tharwat, Hazem M. M. Elmoghazy, Abdelmonem Abdallah

**Affiliations:** 1 Department of Clinical Sciences, College of Veterinary Medicine, Qassim University, Buraidah, Saudi Arabia; 2 Department of Clinical Medicine, University Veterinary Hospital, Qassim University, Buraidah, Saudi Arabia; 3 Department of Health Management, Atlantic Veterinary College, University of Prince Edward Island, Charlottetown, Charlottetown, PE, Canada; 4 Centre for Veterinary Epidemiological Research, Atlantic Veterinary College, University of Prince Edward Island, Charlottetown, Charlottetown, PE, Canada

**Keywords:** camels, diseases, gastrointestinal tract, gut-renal axis, urinary tract, directed acyclic graphs, linear regression models

## Abstract

**Introduction:**

Gastrointestinal (GI) disorders and renal dysfunction are common and clinically significant conditions in dromedary camels (*Camelus dromedarius*). However, their interrelationship—representing the gut–renal axis—remains poorly understood. This study aimed to investigate the association between GI disorders and renal function in camels using biochemical, ultrasonographic, and causal modeling approaches.

**Methods:**

Seventy camels were examined, including 49 with GI disorders and 21 clinically healthy controls. Affected camels were categorized as Group 1 (intestinal obstruction, *n* = 22), Group 2 (GI impaction, *n* = 20), and Group 3 (chronic diarrhea, *n* = 7). Clinical evaluation, ultrasonography of the GI and urinary tracts, and biochemical analyses of serum blood urea nitrogen (BUN) and creatinine were performed. Simple linear regression was used to model associations between GI disorders and renal biomarkers. Directed acyclic graphs were applied to identify potential mediators—hematocrit, total protein, sodium, potassium, albumin, globulin, and phosphorus—and estimate total and direct effects.

**Results:**

Compared with controls, camels in Groups 1–3 had significantly elevated BUN (2.2-, 1.9-, and 2.6-fold) and creatinine (2.4-, 2.7-, and 3.0-fold) concentrations. Adjustment for mediators produced minimal change, suggesting robust and independent associations between GI disorders and renal impairment. Ultrasonographic findings supported these results: intestinal obstruction showed distended loops with reduced motility; impaction exhibited hyperechogenic rumen contents; and chronic diarrhea was associated with mucosal thickening, increased peristalsis, and mesenteric lymphadenopathy.

**Discussion:**

Gastrointestinal disorders in dromedary camels are strongly linked to renal dysfunction, as evidenced by consistent biochemical and ultrasonographic abnormalities. These findings highlight the clinical relevance of the gut–renal axis in camels and emphasize the importance of early recognition and management of GI disorders to prevent secondary renal complications.

## 1 Introduction

Chronic renal disorders (CRDs) are complex and increasingly prevalent conditions affecting dromedary camels, presenting significant challenges in veterinary practice (1–7). Despite their frequency, effective treatment options remain limited and are primarily aimed at managing clinical symptoms and associated complications—such as anemia and abdominal discomfort—rather than reversing disease progression. Current therapeutic approaches include broad-spectrum antimicrobials, anti-inflammatory agents, analgesics, and hematinic preparations (8). Given CRD’s progressive nature and multifactorial etiology, a deeper understanding of its underlying mechanisms is essential to improve both clinical management and long-term outcomes.

Recent advances in veterinary medicine have highlighted a crucial physiological link between the gastrointestinal (GI) system and renal function—commonly referred to as the gut–kidney axis—particularly in small animals such as dogs and cats. This axis plays a key role in the pathogenesis and progression of renal diseases, underscoring the need to explore similar interconnections in large animals, including camels (9).

In human medicine as well as in small animals, a comparable relationship between GI and renal disorders has been proposed (9, 10). Early diagnosis and effective treatment of GI diseases could help delay or even prevent renal complications, including chronic kidney disease (10). Furthermore, interactions between the gut microbiome and kidney function have garnered increasing attention. Intestinal dysbiosis—characterized by microbial imbalance—has been shown to contribute to metabolic disturbances, inflammation, immune suppression, and accumulation of uremic toxins, all of which exacerbate renal dysfunction (11).

In camels, physiological changes in blood urea nitrogen (BUN) and creatinine concentrations have been reported during the last trimester of pregnancy. These changes were attributed to homeorhetic adaptations, whereby camels may divert urea to the GI tract as a source of non-protein nitrogen instead of excreting it in urine (12). However, other studies in camels and goats found no significant fluctuations in BUN and creatinine levels during the three weeks preceding parturition (13, 14).

In research, the primary goal is often to determine whether exposures such as an intervention or risk factor affect or cause an outcome (15). When exposure is linked to an outcome, three explanations are possible: chance (sampling error), bias or confounding that makes unrelated factors appear associated, or a true causal effect (16). Directed acyclic graphs (DAGs), or causal diagrams, are increasingly applied in epidemiology (17). They help identify covariates that should be controlled for, such as confounders in conventional statistical analyses, thereby reducing bias in estimates (18). DAGs also serve as visual tools to represent hypotheses about the causal mechanisms underlying a research question.

Since DAGs are constructed based on researchers’ assumptions, different diagrams can be developed to represent the same research topic (19). This flexibility makes them particularly valuable for analyzing causal relationships, including direct, indirect, and total effects, especially in mediation models where the effect of a putative cause on an outcome may be transmitted through one or more intermediate variables (20).

Using this causal framework, we systematically examined the association between GI disorders and renal dysfunction in dromedary camels by estimating both the total and direct effects. The study aimed to quantify these effects to clarify underlying pathways, thereby supporting earlier diagnosis and potentially improving clinical management in the dromedary camel.

## 2 Materials and methods

### 2.1 Animals

Between January 2015 and May 2025, 70 dromedary camels (*Camelus dromedarius*), aged 3 to 20 years, were examined at the Qassim University Veterinary Hospital. Forty-nine camels presented with various GI disorders, while 21 clinically healthy camels served as controls. The control camels were selected to match the diseased animals in terms of age (±2 years), sex, and general body condition to minimize physiological variability and potential confounding factors. Of the diseased animals, 22 camels (44.9%) were diagnosed with intestinal obstruction (group 1), 20 camels (40.8%) with GI impaction (group 2), and 7 camels (14.3%) with chronic diarrhea (group 3). The duration of illness ranged from 1 to 15 days. Prior to presentation, all diseased camels had received diverse treatments including oral and parenteral antimicrobials, anti-inflammatory agents, analgesics, oral and transrectal mineral oils, intravenous electrolyte and dextrose fluids, antihistamines, and appetite stimulants. The treatment duration varied between 3 and 10 days.

Upon admission, each camel underwent a comprehensive clinical examination, including measurement of respiratory and pulse rates, rectal temperature, assessment of mucous membranes, and auscultation of the thorax and GI tract. All procedures were conducted in accordance with the ethical guidelines approved by the Ethics Committee for the Use and Care of Animals at Qassim University (Buraydah, Saudi Arabia), following the standards outlined in the Guide for the Care and Use of Agricultural Animals in Research and Teaching (21).

### 2.2 Hematological and biochemical analysis

Two jugular blood samples were collected from each animal. The first sample, drawn into EDTA-containing tubes, was used for complete blood count analysis, including leukogram (total white blood cell count and differentials) and hemogram parameters (erythrocyte count, hemoglobin concentration, hematocrit, and red cell indices). The second sample was collected in plain tubes for serum separation. Biochemical analyses included measurements of serum albumin, total protein, globulin, total bilirubin, blood urea nitrogen, creatinine, glucose, calcium, inorganic phosphorus, sodium, and potassium. The enzymatic activity of alkaline phosphatase (ALP) and alanine aminotransferase (ALT) was also assessed.

### 2.3 Ultrasonographic examination

To prepare for GI and renal ultrasonography, the right and left abdominal flanks were clipped and shaved. Sonographic evaluation was performed using 3.5 MHz and 5.0 MHz sector and linear transducers (SonoScape, SonoScape Medical Corp., China). After the application of transmission gel, the second and third gastric compartments, small and large intestines, liver, peritoneum, and right kidney were examined transcutaneously via the right abdominal wall. The caudodorsal ruminal sac, spleen, and left kidney were evaluated from the left paralumbar fossa. Transrectal ultrasonography was used to assess the urinary bladder and left kidney (22–24). In addition, the heart, major vessels, lungs, and pleura were scanned to rule out systemic complications (24–26).

### 2.4 Statistical analysis

Statistical analyses were performed using R version 4.4.2 (R Foundation for Statistical Computing, Vienna, Austria). Differences in the hematological and biochemical findings among the four groups (control, intestinal obstruction, diarrhea, and impaction) were assessed using one-way analysis of variance (ANOVA). When significant differences were detected, pairwise comparisons were performed using Tukey’s honestly significant difference (HSD) *post-hoc* test. Data are expressed as mean ± standard deviation (SD), and a *p*-value of < 0.05 was considered statistically significant.

**Figure 1:**
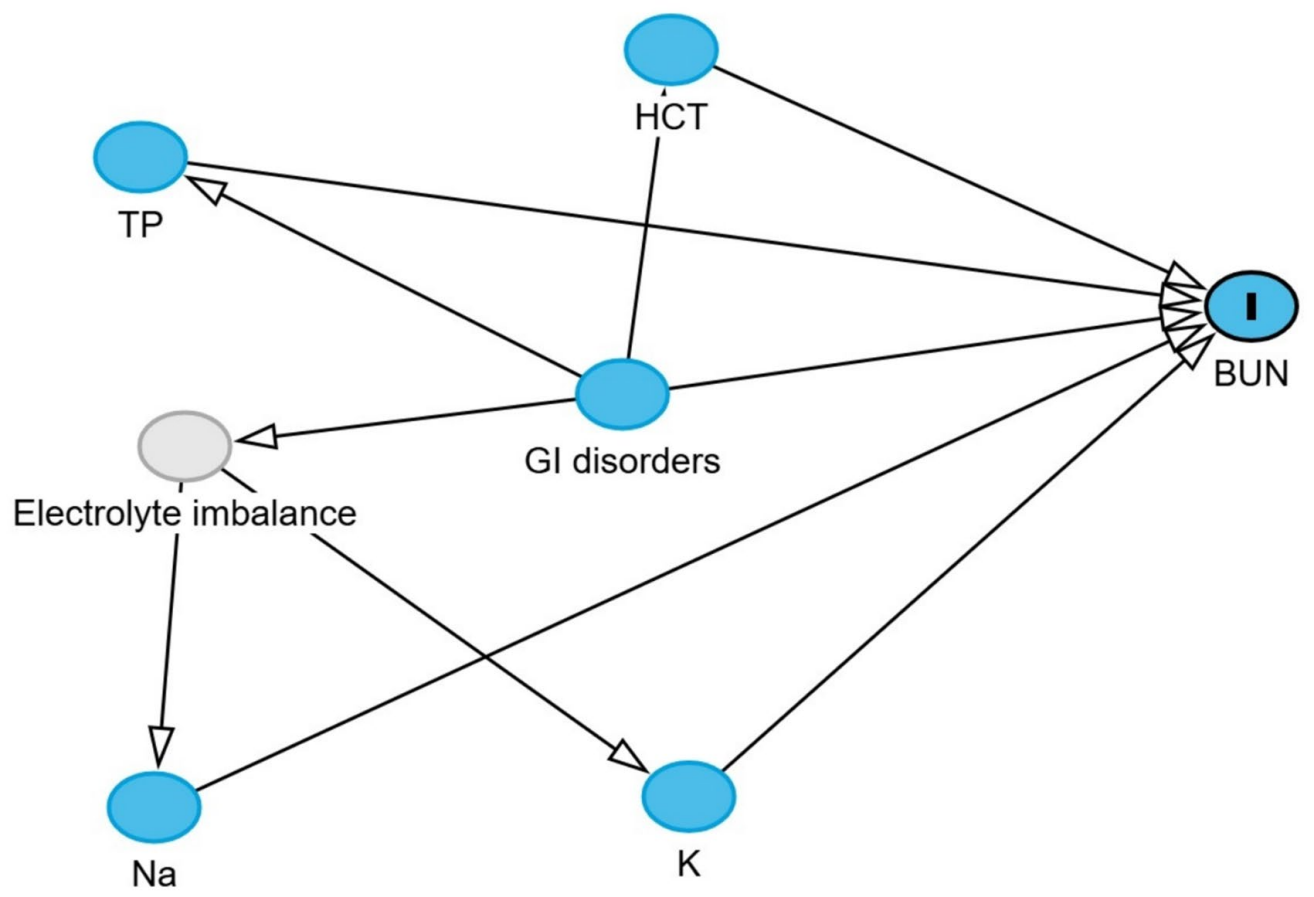
Directed acyclic graph describes the relationship of measured variables to blood urea nitrogen in dromedary camels. BUN: blood urea nitrogen. Ancestors of outcome (blue): gastrointestinal (GI) disorders, total protein (TP), hematocrit (HCT), sodium (Na), and potassium (K). Latent variable (light gray): Electrolyte imbalance.

**Figure 2:**
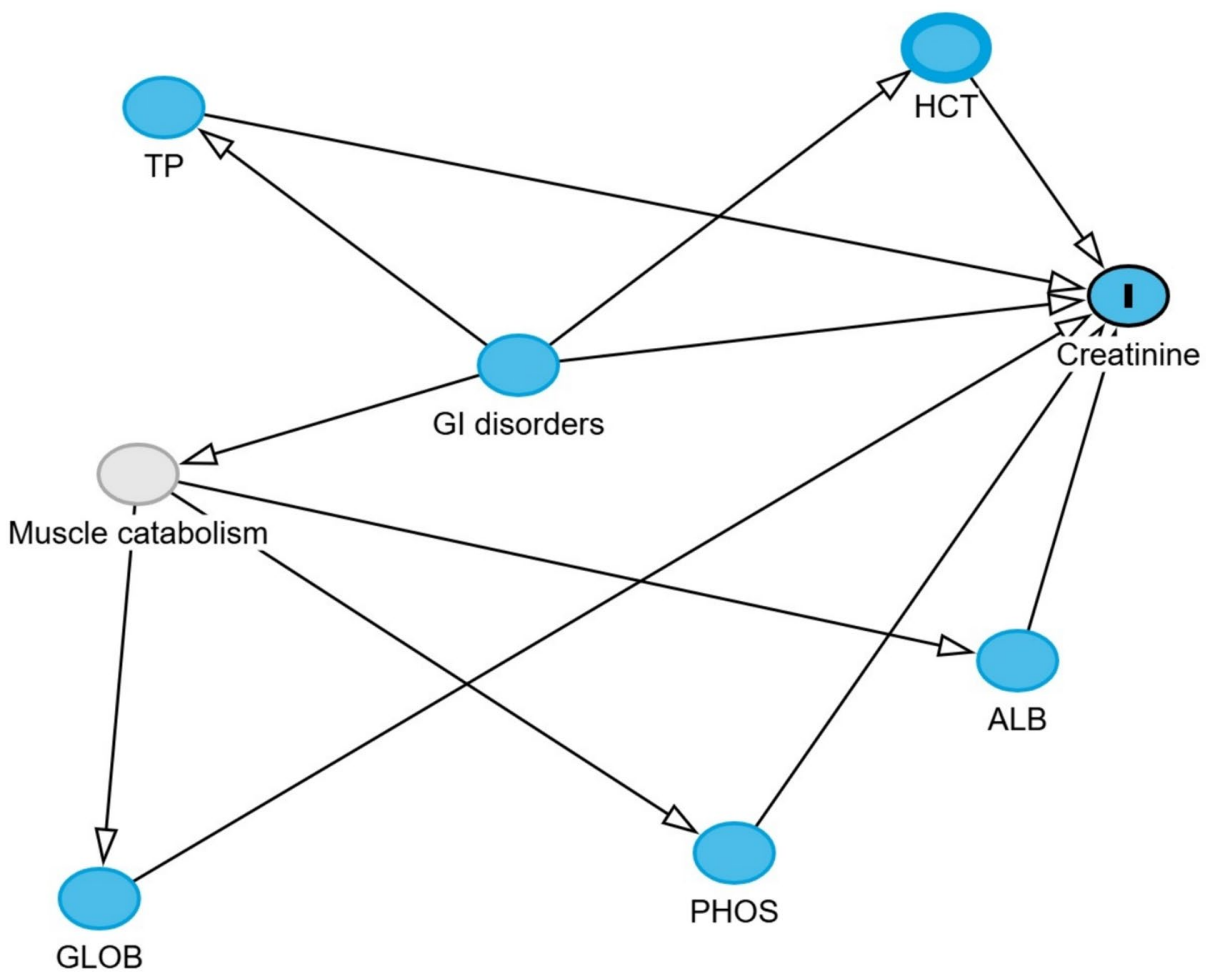
Directed acyclic graph describes the relationship of measured variables to creatinine in dromedary camels. Ancestors of outcome (blue): gastrointestinal (GI) disorders, total protein (TP), hematocrit (HCT), globulin (GLOB), albumin (ALB), and phosphorus (PHOS). Latent variable (light gray): Muscle catabolism.

Directed acyclic graphs (DAGs) were created using the DAGitty^®^ user interface to guide model building, specifically to identify the minimal set of variables required to estimate the total and direct effects of GI disorders on blood urea nitrogen and creatinine levels (27). Additional variables were incorporated into the DAGs based on prior knowledge and plausible biological pathways to account for their potential roles in the exposure–outcome association. These included serum albumin, globulin, total protein, phosphorus, sodium, potassium, and blood hematocrit. Two DAGs, illustrating the outcomes and their relationships with exposure, are presented in Figures 1, 2. Additionally, four DAGs—two for each outcome of interest—were developed to identify the minimally sufficient set of variables to include in the total and direct effect models for each outcome.

The total effect of GI disorders on BUN and creatinine was estimated using linear regression models, with GI disorder status as the main exposure variable. No mediators were adjusted for in these models, so the estimated coefficients reflect the overall impact of GIT disorders on each outcome through both direct and indirect pathways. Any identified pre-exposure confounders were included when present.

The direct effect was estimated by additionally adjusting for mediators identified in the DAGs. For BUN, these were hematocrit, total protein, sodium, and potassium. Adjusting for these variables blocks the indirect pathways, allowing estimation of the association between GIT disorders and BUN that is not mediated through these factors. For creatinine, the mediators were hematocrit, total protein, albumin, globulin, and phosphorus, as identified in the DAGs. To enhance the interpretability of the linear model results, where log-transformed BUN and creatinine were the outcomes, the estimated coefficients were exponentiated and expressed as fold changes in BUN and creatinine concentrations.

## 3 Results

### 3.1 Descriptive statistics

The first diseased group (group 1) consisted of 22 camels (10 females, 12 males) that presented with the absence of defecation lasting 3 to 10 days, along with dehydration, abdominal pain, anorexia, and abdominal distension. The second group (group 2) included 20 camels (11 females, 9 males) that showed clinical signs of gastric impaction, constipation, abdominal pain, inappetence, and vomiting. The third group (group 3) comprised 7 camels (5 females, 2 males) that presented with chronic diarrhea, anorexia, and dehydration. The control group (group 4) consisted of 21 clinically healthy camels (15 females, 6 males) that exhibited no clinical or ultrasonographic abnormalities.

The results of the complete blood count are summarized in Table 1. Camels with intestinal obstruction showed a significant increase in both total leukocyte (*p* = 0.02) and neutrophil counts (*p* = 0.01) compared to controls. Monocyte counts were significantly higher in camels with intestinal obstruction (0.17 ± 0.08 × 10^3^/μL) compared to both the control group and camels with impaction (0.11 ± 0.03 and 0.11 ± 0.06 × 10^3^/μL; *p* = 0.02).

**Table 1:** Descriptive statistics (mean ± SD) of hematological findings in 70 dromedary camels.

Variables	Control (*N* = 21)	Intestinal obstruction (*N* = 22)	Impaction (*N* = 20)	Diarrhea (*N* = 7)
White blood cells (10^3^/μl)	14.1 (3.7)^a^	23.6 (11.8)^b^	15.3 (9.9)^ab^	23 (9.54)^ab^
Lymphocytes (10^3^/μl)	2.05 (1.44)	1.37 (0.67)	1.35 (0.78)	1.18 (1.08)
Monocytes (10^3^/μl)	0.11 (0.03)^a^	0.17 (0.08)^b^	0.11 (0.06)^a^	0.15 (0.06)^ab^
Neutrophils (10^3^/μl)	10.9 (3.84)^a^	20.9 (11.4)^b^	13 (9.65)^ab^	20.7 (9.39)^ab^
Eosinophils (10^3^/μl)	1.09 (0.87)	1.16 (1.44)	0.84 (0.65)	0.97 (0.45)
Red blood cells (10^6^/μl)	11.3 (1.44)	58.3 (220)	11.8 (2.79)	11.1 (2.47)
Hemoglobin (g/dl)	13.9 (1.49)^a^	16.6 (3.55)^b^	15.8 (4.16)^ab^	13.9 (3.27)^ab^
Hematocrit %	29.9 (3.82)	31.1 (7.01)	31.6 (7.74)	28 (4.67)
Mean Corpuscular Volume (fl)	26.8 (1.89)	27 (2.38)	26.7 (2.43)	25.9 (1.86)
Mean Corpuscular Hemoglobin (pg)	12.4 (1.2)^a^	14.5 (2.01)^b^	13.2 (1.47)^ab^	12.6 (0.84)^a^
Mean Corpuscular Hemoglobin Concentration (g/dl)	46.8 (3.89)^a^	52.9 (7)^b^	49.6 (4.07)^ab^	49.2 (4.1)^ab^

Within each row, means with different superscripts differ significantly at a *P*-value of < 0.05.

Hemoglobin levels were significantly higher in camels with intestinal obstruction (16.6 ± 3.55 g/dL) compared to the control group (13.9 ± 1.49 g/dL; *p* = 0.02). Both mean corpuscular hemoglobin and mean corpuscular hemoglobin concentration were significantly increased in camels with intestinal obstruction relative to the control group (*p* = 0.001 and *p* = 0.02, respectively). No statistically significant differences were noted among the groups in lymphocyte or eosinophil counts, red blood cell counts, hematocrit, or mean corpuscular volume.

**Figure 3:**
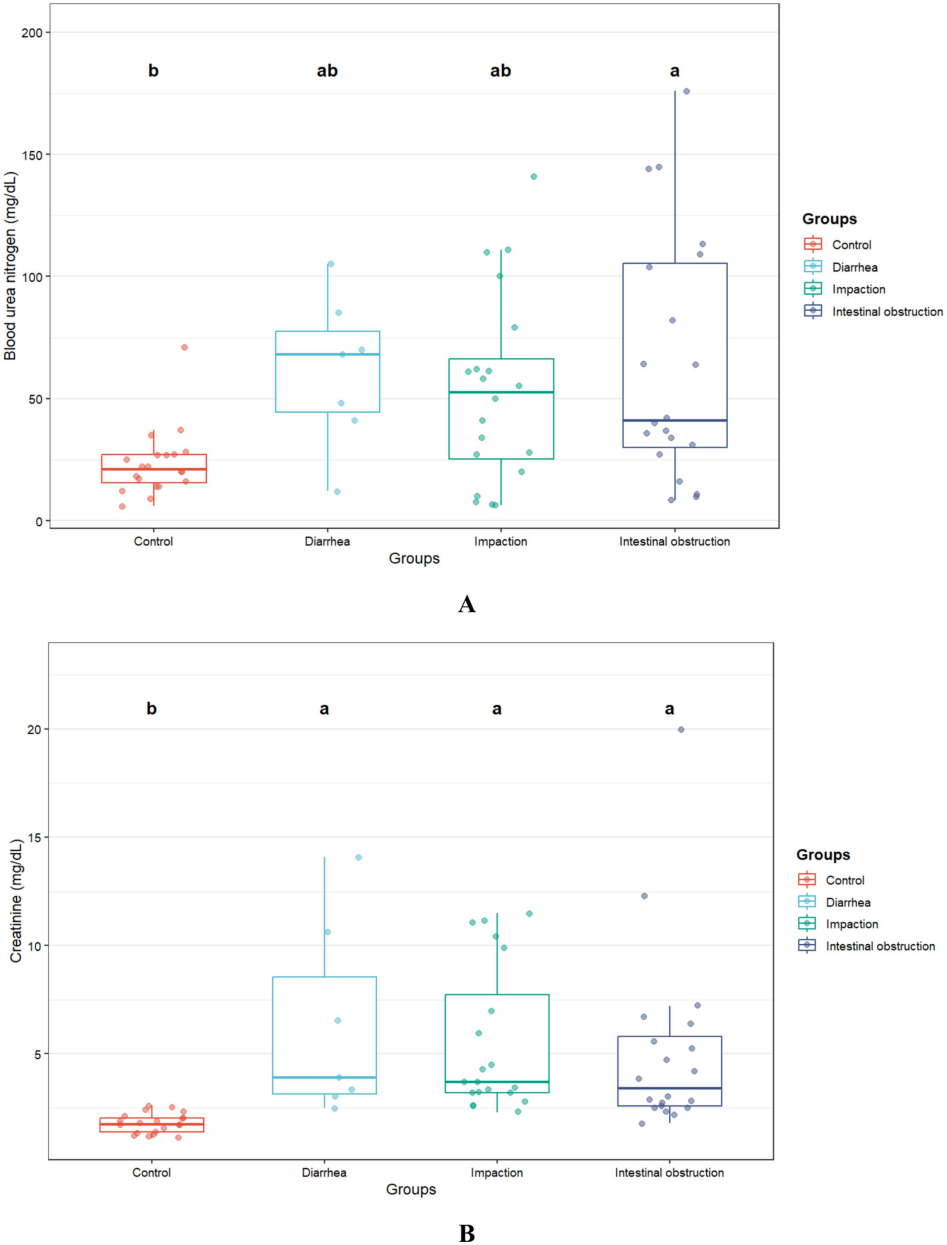
Blood urea nitrogen **(A)** and creatinine **(B)** concentrations in camels with diarrhea (*n* = 7), gastric impaction (*n* = 20), and intestinal obstruction (*n* = 23) compared to healthy controls (*n* = 21). Different superscript letters indicate significant differences (*p* < 0.05).

The biochemical results are presented in Table 2. Blood urea nitrogen levels were significantly elevated in camels with intestinal obstruction (64.6 ± 50.8 mg/dL), compared to the control group (23.1 ± 13.5 mg/dL; *p* = 0.004). Serum creatinine levels were also markedly elevated across all diseased groups (*p* = 0.001), exceeding three times the average values observed in healthy controls (Figure 3). Amylase levels were significantly higher in camels with intestinal obstruction and diarrhea compared to controls (*p* = 0.01). No significant intergroup differences were observed for serum albumin, ALP, ALT, total bilirubin, calcium, phosphorus, glucose, sodium, potassium, total protein, or globulin.

**Table 2:** Descriptive statistics (mean ± SD) of biochemical findings in 70 dromedary camels.

Variables	Control (*N* = 21)	Intestinal obstruction (*N* = 22)	Impaction (*N* = 20)	Diarrhea (*N* = 7)
Albumin (g/dl)	5.54 (8.18)	3.97 (1.07)	3.65 (1.01)	2.96 (0.9)
Alkaline phosphatase (U/l)	52.2 (18.9)	85.9 (98.4)	80.4 (68.1)	66.4 (61.8)
Alanine aminotransferase (U/l)	13.3 (5.87)	63.1 (168)	23.4 (29.6)	20.9 (13.1)
Amylase (U/l)	1,020 (157)^a^	1,380 (373)^b^	1,180 (351)^ab^	1,530 (726)^b^
Total bilirubin (g/dl)	0.29 (0.05)	0.34 (0.08)	0.32 (0.07)	0.3 (0.05)
Calcium (g/dl)	10 (0.58)	17.3 (17.7)	20.3 (27)	10.1 (1.27)
Phosphorus (g/dl)	5.88 (1.99)	8.64 (3.35)	11.6 (14.5)	7.86 (4.52)
Blood Urea Nitrogen (g/dl)	23.1 (13.5)^a^	64.6 (50.8)^b^	53.4 (38.8)^ab^	61.3 (30.6)^ab^
Creatinine (g/dl)	1.76 (0.45)^a^	5.07 (4.31)^b^	5.49 (3.35)^b^	6.27 (4.46)^b^
Glucose (g/dl)	146 (33.2)	174 (70.8)	193 (113)	170 (45.2)
Sodium (mmol/l)	146 (5.26)	149 (7.28)	148 (8.44)	142 (7.45)
Potassium (mmol/l)	4.68 (0.49)	4.19 (1.12)	4.09 (1.35)	4.69 (0.8)
Total Proteins (g/dl)	6.98 (0.89)	10.9 (15.4)	6.96 (1.16)	6.31 (1.55)
Globulin (g/dl)	3.2 (0.87)	3.54 (1.2)	3.31 (0.87)	3.36 (1.05)

Within each row, means with different superscripts differ significantly at a *P*-value of < 0.05.

### 3.2 Model results

In the unadjusted model (total effect), intestinal obstruction, impaction, and diarrhea were associated with mean BUN levels that were 2.2-fold (*p* = 0.002), 1.9-fold (*p* = 0.01), and 2.6-fold (*p* = 0.01) higher, respectively, compared to the control group. After controlling for hematocrit, serum potassium, sodium, and total protein (direct effect), the corresponding mean ratios were 2.64 (*p* < 0.001), 2.05 (*p* = 0.008), and 2.44 (*p* = 0.01). Both models are presented in Table 3.

**Table 3:** Total and direct effects of gastrointestinal disorders on blood urea nitrogen levels.

Variables	Total effect			Direct effect		
	Estimate	Standard error	*p*-value	Estimate	Standard error	*p*-value
GI disorders						
Control	Ref					
Intestinal obstruction	0.81	0.25	**0.002**	0.97	0.26	**<0.001**
Impaction	0.61	0.25	**0.01**	0.71	0.26	**0.008**
Diarrhea	0.93	0.35	**0.01**	0.89	0.35	**0.01**
Hematocrit				−0.02	0.01	0.1
Total protein				−0.01	0.01	0.1
Sodium				0.003	0.01	0.8
Potassium				0.01	0.01	0.3

In the unadjusted model assessing the total effect, intestinal obstruction, impaction, and diarrhea were each associated with significantly higher creatinine levels. Specifically, intestinal obstruction was associated with a 2.4-fold increase (*p* < 0.001), impaction with a 2.7-fold increase (*p* < 0.001), and diarrhea with a 3.0-fold increase (*p* < 0.001) compared to the control group. After controlling for hematocrit, total protein, phosphorus, globulin, and albumin, the direct effects remained largely unchanged: intestinal obstruction (2.3-fold increase, *p* < 0.001), impaction (2.6-fold increase, *p* < 0.001), and diarrhea (3.0-fold increase, *p* < 0.001). Table 4 presents the results of both models. None of the blood parameters included in the models showed significant associations with the outcomes.

**Table 4:** Total and direct effects of gastrointestinal disorders on creatinine levels.

Variables	Total effect			Direct effect		
	Estimate	Standard error	*P*-value	Estimate	Standard error	*p*-value
GI disorders						
Control	Ref					
Intestinal obstruction	0.87	0.16	**<0.001**	0.84	0.17	**<0.001**
Impaction	1.01	0.16	**<0.001**	0.96	0.17	**<0.001**
Diarrhea	1.1	0.22	**<0.001**	1.1	0.23	**<0.001**
Hematocrit				0.01	0.01	0.1
Total protein				−0.007	0.008	0.3
Albumin				−0.004	0.01	0.7
Globulin				0.01	0.07	0.8
Phosphorus				0.001	0.008	0.8

### 3.3 Ultrasonographic findings

**Figure 4:**
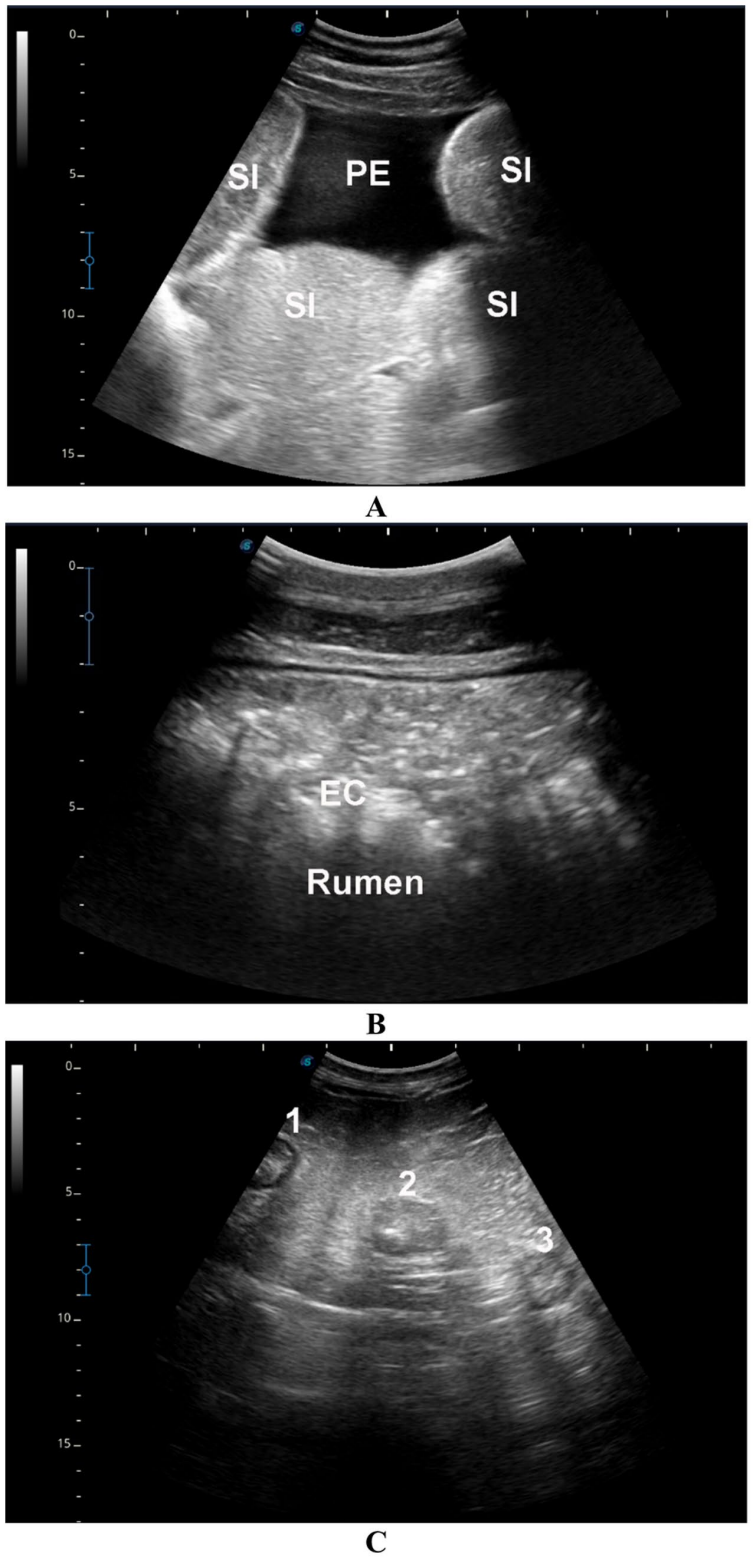
Ultrasonographic findings in camels with gastrointestinal disorders. **(A)** Distended small intestinal loops (SI) with mild echoic peritoneal effusion (PE) in a male camel with intestinal obstruction. **(B)** Echogenic rumen contents (EC) in a male camel with rumen impaction. **(C)** Enlarged mesenteric lymph nodes (labeled 1, 2, 3) in a female camel with a 7-day history of diarrhea.

In camels with intestinal obstruction (group 1), ultrasonographic examination revealed markedly distended intestinal loops with either reduced or absent motility in 17 out of the 22 animals (77.3%). Peri-intestinal fluid accumulation was observed in 20 camels (90.9%) appearing hypoechoic, and in 2 camels (9.1%) appearing echogenic. In the impaction group (group 2), rumen contents were not visualized in 15 of the 20 camels (75%), while in the remaining 5 animals (25%), the contents appeared subjectively hyperechogenic. All camels with diarrhea (group 3) exhibited increased peristaltic activity. Among these, four camels (57.1%) showed sonographic thickening of the intestinal mucosa, while the remaining three camels (42.9%) had visibly enlarged mesenteric lymph nodes (Figure 4). None of the control camels (group 4) showed any abnormalities on GI or urinary tract ultrasonography.

## 4 Discussion

In camels with intestinal obstruction, the clinical presentation was dominated by anorexia, abdominal distension, absence of feces for 3 to 10 days, dehydration, and abdominal pain. These findings align closely with previous reports on dromedary camels (28–31). Camels presenting with gastric impaction primarily showed inappetence, constipation, vomiting, and episodic abdominal pain—signs consistent with those described in cases of gastric distension (28, 32). Those with diarrhea were admitted with anorexia, dehydration, and chronic diarrhea, symptoms comparable to chronic enteritis in camels (33).

Regarding hematological changes, neutrophilic leukocytosis was evident in camels with intestinal obstruction compared to healthy controls. Monocytosis was also observed in camels with intestinal obstruction relative to those with impaction and controls. Additionally, hemoglobin concentration, mean corpuscular hemoglobin, and mean corpuscular hemoglobin concentration were significantly elevated in camels with intestinal obstruction. These hematological alterations reflect patterns previously documented in camels with abdominal distension and chronic watery diarrhea (28, 33). Biochemically, most parameters did not differ significantly among groups except for blood urea nitrogen and creatinine, both of which were markedly elevated in all camels with GI disorders compared to healthy controls. Similar biochemical profiles have been reported in camels suffering from abdominal distension and chronic diarrhea (28, 33).

Both the total and direct effect models indicate that intestinal obstruction, impaction, and diarrhea are associated with elevated BUN and creatinine levels. In the unadjusted models, these GI conditions showed strong associations with both outcomes, reflecting their total effects. After controlling hematocrit, serum electrolytes, and protein parameters, the associations remained largely unchanged, suggesting that the observed effects are primarily driven by the GI conditions themselves rather than by the included biochemical covariates. These findings highlight a robust and independent relationship between GI disturbances and renal function markers, emphasizing the importance of monitoring BUN and creatinine in affected individuals. Comparable associations between GI and renal dysfunction have been reported in human medicine (10). Alterations in the gut microbiome play a significant role in kidney disease, as intestinal dysbiosis can disrupt microbial balance, leading to inflammation, metabolic disturbances, immune dysregulation, and accumulation of uremic toxins—all factors that may accelerate the progression of renal failure (11).

Mediation analysis assesses whether and how the effect of exposure on an outcome operates through intermediate variables by decomposing the total effect into direct and indirect components (20). In this study, we identified potential mediators to include in a given model, using the DAGs (17). The DAGs have long been used as an informal tool for causal analysis (34). Traditional approaches to addressing confounding bias through automated model-building were shown to be problematic, often leading to inadequate analyses and misleading interpretations (18). In contrast, DAGs provide a clear and transparent framework for identifying and illustrating assumptions about causal relationships among variables, but they must be carefully constructed to avoid incorrect causal pathways or the omission of important confounders that could introduce bias (35).

Ultrasonography has proven to be an effective diagnostic modality in dromedary camel medicine, enabling evaluation of normal thoracic and abdominal organs as well as detection of various intra-abdominal pathologies (1–5, 7, 8, 22–26, 28, 30–33, 36–45). In this study, ultrasound in camels with intestinal obstruction revealed markedly distended intestines with significantly reduced or absent motility, accompanied by hypoechoic to echogenic peri-intestinal fluid consistent with prior findings (28). In camels with gastric impaction, rumen contents were often difficult to visualize and appeared hyperechoic in approximately one-quarter of cases, findings in agreement with reports of rumen impaction secondary to pica or depraved appetite (32). Camels with diarrhea showed increased intestinal peristalsis, mucosal thickening, and enlarged mesenteric lymph nodes, similar to the changes reported in camels with paratuberculosis (33).

Although this study did not investigate gut microbiome alterations—an acknowledged limitation—further research is warranted to elucidate the pathophysiological mechanisms linking GI disorders and renal dysfunction in camels. We hypothesize that digestive disorders, particularly intestinal obstruction and impaction, may impair renal perfusion due to increased intra-abdominal pressure exerted on major abdominal vessels, including the abdominal aorta and renal arteries, potentially leading to renal insufficiency. This hypothesis is supported by analogous observations in late-pregnant camels, where renal dysfunction may result from fetal compression of renal vasculature (12). Future studies should also incorporate gut microbiome profiling to explore additional microbial-mediated pathways within the gut–kidney axis, which may further clarify the interplay between GI and renal health in dromedaries. Given the growing recognition of the gut microbiome as a key regulator of systemic health, future studies should also aim to investigate microbial composition and diversity in dromedary camels with GI and renal disorders. Integrating microbiome profiling into this line of research may uncover important microbial signatures or dysbiosis patterns that contribute to the pathophysiology of the gut–kidney axis, thereby enriching our understanding of the underlying mechanisms.

One of the main limitations of this study is the absence of follow-up data on the clinical progression of the camels after medical or surgical treatment. As such, it remains unclear whether the observed renal dysfunction is reversible or persistent over time. This limits the clinical interpretability of the findings. Future longitudinal studies are warranted to monitor the long-term renal and GI outcomes in affected animals and to better understand the dynamics of this gut–kidney interplay. An additional limitation of this study lies in the variability of therapeutic interventions administered to diseased camels prior to their admission. The use of antimicrobials, anti-inflammatories, electrolyte solutions, and other agents may have affected renal biomarkers such as serum creatinine, BUN, and electrolyte levels, potentially confounding the interpretation of renal function in relation to GI disease. Although treatment histories were documented, the diversity in medication types, dosages, and durations introduced a level of heterogeneity that could not be fully controlled. Thus, future studies should consider evaluating untreated animals or establishing standardized treatment protocols prior to sampling to better isolate the pathophysiological link between GI and renal disorders.

## 5 Conclusion

This study establishes a strong and independent association between GI disorders and renal dysfunction in dromedary camels, evidenced by elevated BUN and creatinine levels and supported by ultrasonographic findings. These relationships persisted even after adjusting for relevant biochemical mediators, suggesting a direct link between GI pathology and renal impairment. The findings underscore the clinical importance of early diagnosis and intervention in GI disorders to prevent secondary renal complications. Although limitations such as treatment variability and lack of follow-up exist, this study highlights the need for standardized protocols and future longitudinal and microbiome-focused studies to better understand the gut–kidney axis in camels and improve clinical outcomes.

## Data Availability

The original contributions presented in the study are included in the article/supplementary material, further inquiries can be directed to the corresponding author.
